# Supplementary material for: Electrochemical and Fluorescent Properties of Crown Ether Functionalized Graphene Quantum Dots for Potassium and Sodium Ions Detection
Source: Nanomaterials (Basel). 2021 Oct 29;11(11):2897. doi: 10.3390/nano11112897 (PMC8625964; doi:10.3390/nano11112897)
Supplement: Supplementary file 1 [file nanomaterials-11-02897-s001.zip › nanomaterials-1435552-supplementary.pdf]

Supplementary Materials

to

Electrochemical and fluorescent properties of crown ether functionalized graphene quantum dots for potassium and sodium ions detection

Daniela Iannazzo,[1,*] Claudia Espro,[1*] Angelo Ferlazzo,[1] Consuelo Celesti,[1] Caterina Branca[2] and Giovanni Neri[1]

[1] *Department of Engineering, University of Messina, Contrada Di Dio, 98166 - Messina, Italy; espro@unime.it (C.E.);angelo.ferlazzo@unime.it (A.F.);ccelesti@unime.it (C.C.);gneri@unime.it (G.N.)*

[2] *Department of Mathematical and Computer Sciences, Physical Sciences and Earth Sciences, University of Messina, I-98166 Messina, Italy; cbranca@unime.it (C.B.)*

* *Correspondence: diannazzo@unime.it; espro@unime.it; Tel.: +39 0906765569*

Table of Contents

Figure S1. EIS measurements for GQDs-18-crown-6/SPCE, GQDs /SPCE, bare SPCE.......S2

Figure S2. Reliability of GQDs-18-crown-6 based electrode in different measurements... S2

Figure S3. PL spectra of GQDs, NH_2-15-crown-5 and NH_2-18-crown-6 in the presence of different concentrations of NaCl or KCl...S3

Figure S4. PL spectra of GQDs-18-crown-6 in the presence of NaCl 100 mM, KCl 100 mM or in the presence of KCl and NaCl, 100 mM...................S4

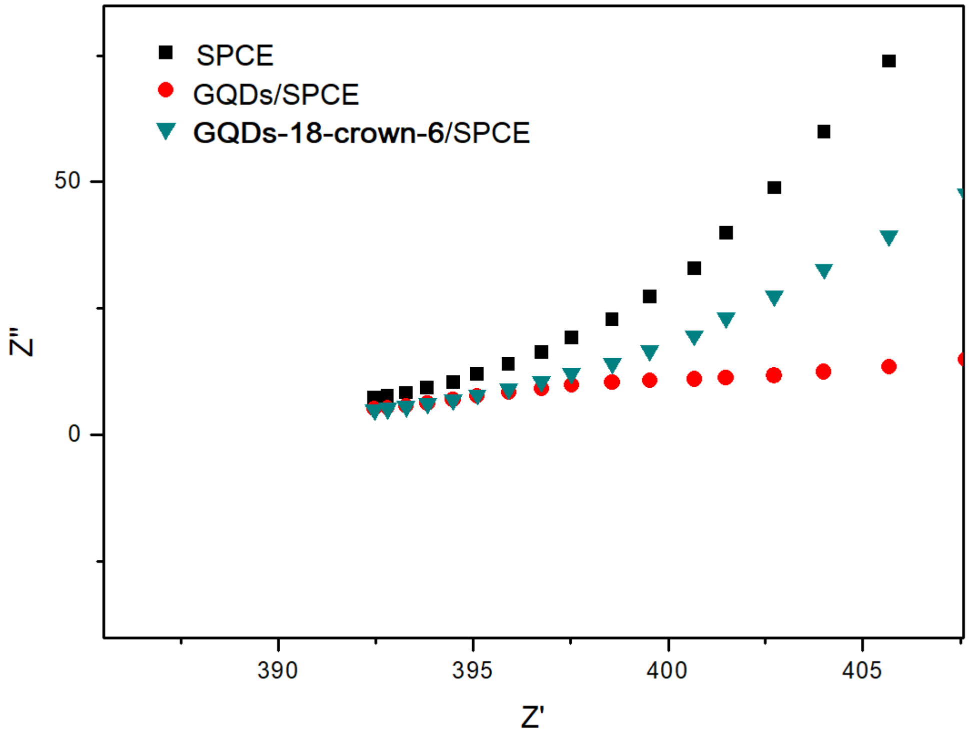

Figure S1. EIS measurements for GQDs-18-crown-6/SPCE, GQDs /SPCE and bare SPCE.

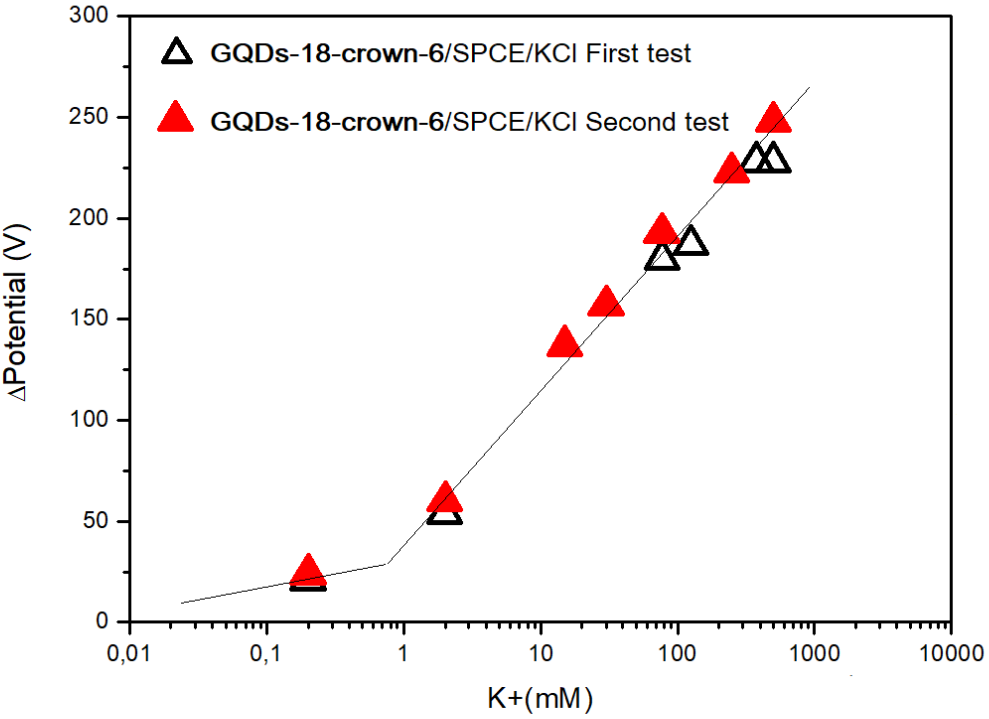

Figure S2. Reliability of the GQDs-18-crown-6 based electrode response in two different measurements.

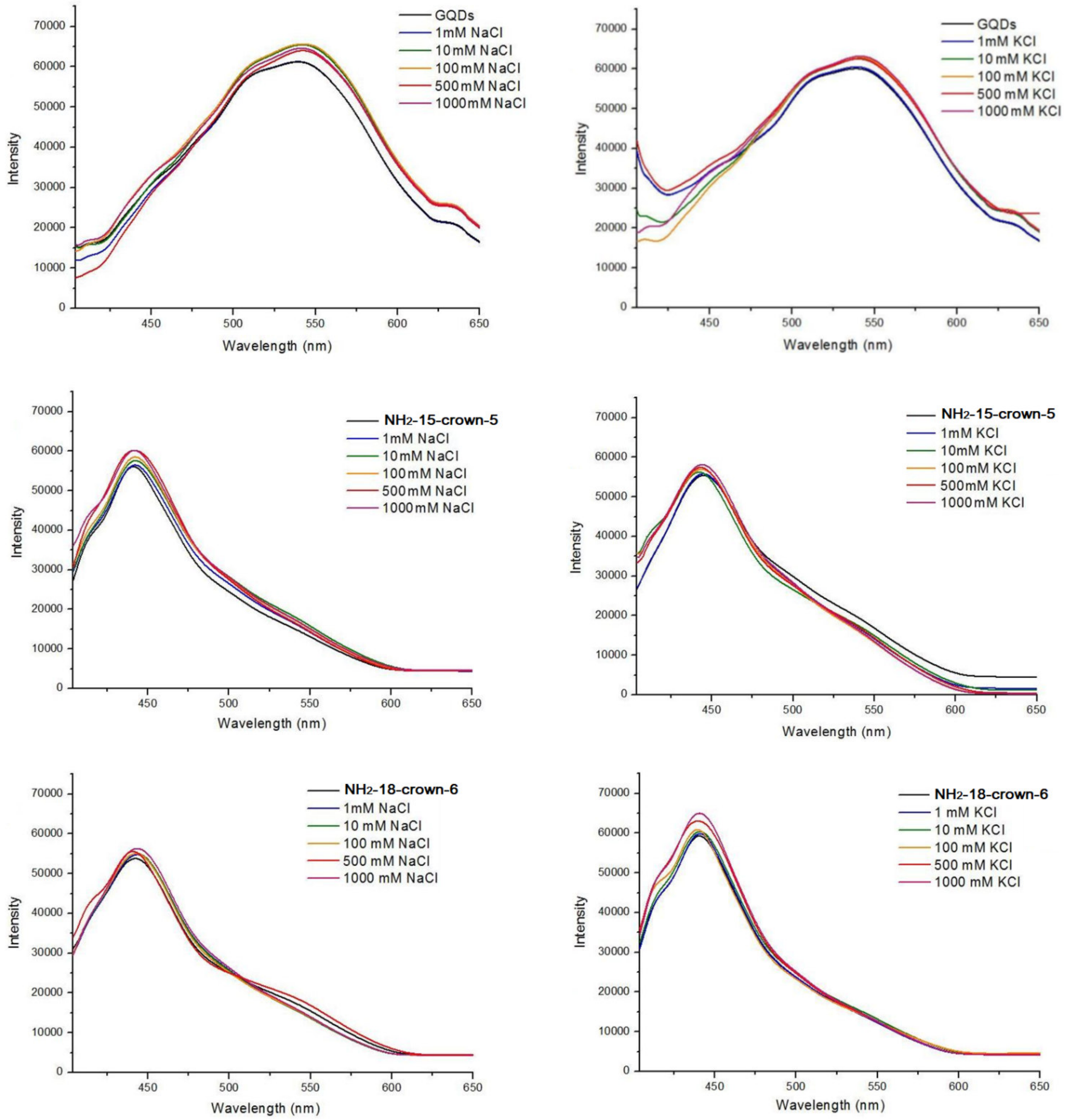

Figure S3. PL spectra of: GQDs in the presence of different concentrations of (a) NaCl or (b) KCl, NH_2-15-crown-5 in the presence of different concentrations of (c) NaCl or (d) KCl, NH_2-18-crown-6 in the presence of different concentrations of (e) NaCl or (f) KCl. All spectra were performed at the λ_{exc} of 360 nm at a concentration of 100 ng/mL.

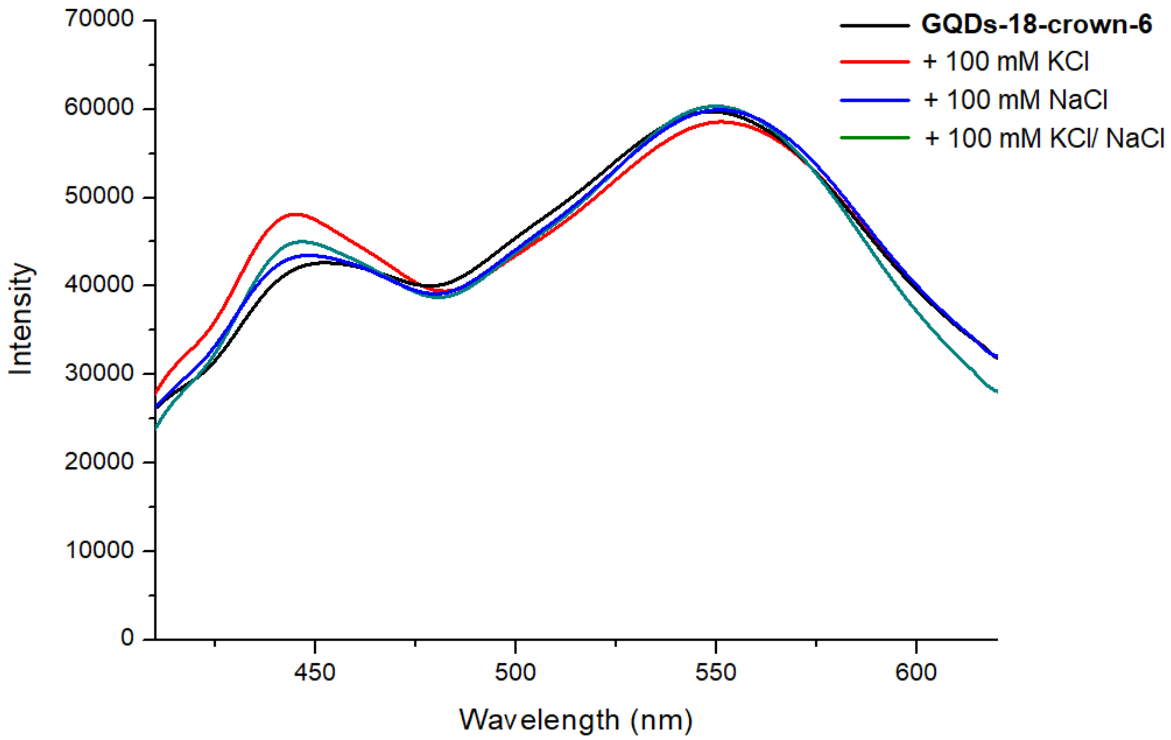

Figure S4. PL spectra of GQDs-18-crown-6 and of the same sample in the presence of NaCl 100 mM, KCl 100 mM or in the presence of KCl and NaCl, 100 mM. All spectra were performed at the λ_{exc} of 360 nm at a concentration of 100 ng/mL.
